# Giving Microbes Their Due

**DOI:** 10.1371/journal.pbio.1000425

**Published:** 2010-07-20

**Authors:** Norman R. Pace

**Affiliations:** Department of Molecular, Cellular and Developmental Biology, University of Colorado, Boulder, Colorado, United Stated of America

**Figure pbio-1000425-g001:**
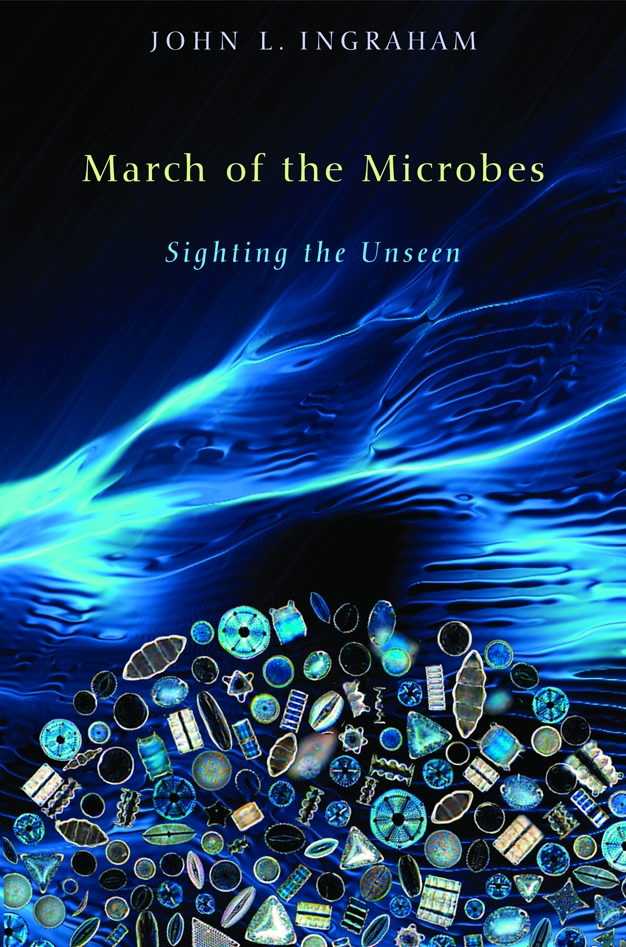
Ingraham JL (2010) March of the Microbes: Sighting the Unseen. **Cambridge, MA: Harvard University Press. 326p. ISBN 978-0-674-03582-9. US$29.95.**

[Fig pbio-1000425-g001]Most of life's diversity springs from microbial organisms, which carry out the lion's share of the chemical transformations that drive the biosphere. It is therefore remarkable that the microbial world is so little recognized by the general public except in the context of disease and rot. Unfortunately, general biology textbooks at all educational levels do little to rectify this taxonomic snub, as most give short shrift to the remarkably diverse multitudes of microbes that live among us.

The reason for the nearly universal neglect of microorganisms is obvious: humans cannot see the individual organisms—the microbes—without a microscope. Instead, perception of the microbial world with our normal senses usually requires observation of the consequences of microbial activities, what John Ingraham refers to as “sightings” of microbes in his new book, *March of the Microbes: Sighting the Unseen*. Ingraham intends to bring some view of the importance and ubiquity of the microbial world to a general audience, a task for which he is well qualified. Ingraham, a former president of the American Society for Microbiology and UC Davis professor emeritus of microbiology, has long been a prominent microbiologist and textbook author. He also has accumulated over his long career a large fund of anecdotes—sightings—of the microbial world, many recounted here.

Following a brief introduction to microbes and the microbial way of life—including an admonishment to the reader who dares to dismiss our microbial progenitors that humans are the “recent intruders into their well-established and self-developed world”—the author ambles through a succession of macroscopic manifestations of microbial activities, past and present. The descriptions are grouped sensibly. For instance, in the context of foods (the chapter “Food and Drink”) in addition to many interesting tales, we learn fun facts about wine and yeast—such as, “Winemakers like to say that they calculate the amount of sugar needed to reach the ideal pressure in the [champagne] bottle. But, in fact, the yeast makes this decision.”—the multiple antimicrobial properties of the unrefrigerated egg, and the microbiology behind additives such as the high fructose corn syrup and xanthan gum that lace much of our readily available foods. (Read the labels!) Did you know that “xanthan gum” is just a killed culture of *Xanthomonas campestris*? This organism produces copious amounts of an extracellular capsule that happens to have qualities useful in food manufacture (it's often added to salad dressings and sauces as a thickening agent).

Other chapters touch on interesting symbioses (“Living Together,” which Ingraham notes “is a characteristic pattern of microbial life), environmental issues all around us (“Cycling Carbon,” in which the fate of plastic reveals human perturbation of Earth's carbon cycle as well as potential utilities of microbes), extremophiles (“Hostile Environments,” from “blistering temperatures and corrosive acidity” to “crushing hydrostatic pressure”), pathogens and their therapy (“Felonius Bacteria,” which have “no notion of working for the common good”), and past and present impacts of microbes on the world around us (“Shapers of the Planet,” from oxygen-evolving cyanobacteria to antibiotic-producing actinomycetes). With such rich examples of the persistence and ubiquity of microbial processes, these, and still other chapters, invoke many different “sightings” of microbial activities, all interesting and with greater or lesser impact on the biosphere and humans. Simple chemical explanations, mainly done with text, are used to interpret some of the descriptions, but lack of chemical knowledge does not detract much from understanding the phenomena.

This small volume (only 5 1/2“×8”) is almost all text. It contains no photographs and relatively few diagrams (which are generally well done, simple, and to the point). At first the low level of illustration surprised me, although, with the World Wide Web at hand, any answers to auxiliary questions the text might elicit are at hand with a keystroke.

Overall, I enjoyed this book considerably and would recommend it to anyone with a passing interest in learning about the microbial world. It would also serve as a useful resource for teachers. I culled several of the “sightings” for use in my own microbiology course, and I suspect that most teachers of microbiology also would find some useful nuggets for their own courses.

All that said, my enthusiasm for the book was tempered by a rather outdated portrayal of microbial diversity. For instance, Ingraham offers a good explanation of the ribosomal RNA-based universal phylogenetic tree, and he acknowledges the vast differences between archaea and bacteria. But then he conflates the two into “prokaryote,” characterized by their lack of internal architecture. At this stage of understanding of phylogenetic diversity and cell structure, I believe it is a disservice to students to continue to pander to the historical artifact that “prokaryote” has certainly become, with its simplistic division of life forms based on putative organizational structure. Further, we are informed, “Bacteria are divided into two major groups—Gram positive and Gram negative.” This traditional staining protocol perhaps is a useful simplification for describing the few organisms with medical relevance, but it has no phylogenetic significance and certainly is no modern criterion with which to describe microbial diversity.

In short, Ingraham's new book is an easy-to-read and generally informative introduction to the microbial world that surrounds us. And he rightly points out that microbes are far better equipped to survive any natural or human-wrought calamity that may lie ahead. But his descriptions of the microbes themselves, and microbial diversity I found a bit retro.

